# Colonic Involvement in a Patient with Chronic Lymphocytic Leukaemia

**DOI:** 10.1155/2008/742146

**Published:** 2008-06-19

**Authors:** P. E. T. Arkkila, H. Nuutinen, F. Ebeling, E. Elonen, P. Kärkkäinen, M.-L. Karjalainen-Lindsberg

**Affiliations:** ^1^Department of Gastroenterology, Helsinki University Central Hospital, 00290 Helsinki, Finland; ^2^Department of Haematology, Helsinki University Central Hospital, 00290 Helsinki, Finland; ^3^Department of Pathology, Helsinki University Central Hospital, 00290 Helsinki, Finland

## Abstract

Various gastrointestinal infiltrations have been described in patients with chronic lymphocytic leukaemia (CLL). Here, we report a 69-year-old man with CLL and anaemia in whom the macroscopic finding of colonoscopy was normal, but the histological specimens revealed lymphocytic leukemia in ileum and in colon. If a CLL patient has any symptoms suggesting a possible GI manifestation of the haematologic disease or anaemia not explained by bone marrow infiltration or hemolysis, the diagnostic evaluation should include endoscopies with adequate biopsies.

## 1. INTRODUCTION

Gastrointestinal manifestations have only very rarely been described in patients with chronic lymphocytic leukaemia (CLL). The lymphocytic infiltration seems to depend on the tumour burden and proliferation activity [1]. Conventional staging according to Rai or Binet may not accurately reflect the whole extent of the disease. It is not known if CLL patients should possibly be candidates for an endoscopic investigation and whether proof of gastrointestinal involvement would influence the treatment decisions.

We present a CLL patient who underwent gastrointestinal endoscopies because of anaemia and who was found to have colonic histological CLL manifestations in spite of normal macroscopic appearance of the mucosa.

## 2. CASE REPORT

The patient is a 69-year-old-man, an exsmoker with chronic atrial fibrillation and chronic otitis and warfarin and digitalis as his only medications. His past medical history was unremarkable until the end of 2001, when he started to have productive cough and was found to have mild leukocytosis. In 2002, the plain chest x-ray was first considered to be suggestive of sarcoidosis. The CT scan showed lymphadenopathy (max. 2 cm) in both pulmonary hilar areas, in the axillae, and also below the diaphragm, the ultrasound examination also in the jugular and supraclavicular areas. The pathological examination of mediastinoscopic paratracheal and subcarinal lymph node biopsies showed effacement of the normal lymph node architecture by infiltration of small, CD5+, CD20+, and CD23+ lymphatic cells with vague pseudofollicular organization consistent with the diagnosis of small B-cell lymphocytic lymphoma/CLL. Neither CD38 nor overexpression of p53 was observed by immunohistochemistry, but the lymphoma cells expressed ZAP-70 as an adverse prognostic factor.

In 2002, the haemoglobin level was 133–139 g/L, the white cell count 11.0–12.9 × 10E9/L with 59% lymphocytes, the platelet count 191–289 × 10E9/L, and the Coombs test negative. Elevated levels of the plasma lactate dehydrogenase activity, 967 U/l (normal below 450 U/l), and the serum thymidine kinase activity, 34 U/l (normal below 8 U/l), were found. The bone marrow aspirate showed a decreased proportion of erythropoiesis as well as granulopoiesis and an increased proportion of small mature lymphocytes, up to 80–90% of the cellularity.

The bone marrow biopsy showed 60% overall cellularity. 60% of cells were interstitially and diffusely between trabeculae, infiltrating small lymphocytes admixed with scarce prolymphocytes and paraimmunoblasts. The immunophenotype was identical to the lymph node infiltrate.

No treatment was considered necessary, and the patient'S clinical condition remained stable during 2002-2003. In the follow up CT'S the size of lymph nodules remained similar. The cough had disappeared, and the patient did not have any general symptoms such as fever, abnormal sweating, or weight loss. A small two-component IgG kappa serum paraprotein (2.6 g/L + 1.0 g/L) was detected. In 2004, after a left-sided pneumonia, slight progression of the lymphadenopathy in the left pulmonary hilus, para-aortal, and mesenterial regions was found without splenomegaly. In spite of that, as well as the appearance of mild night sweats, treatment was not considered necessary, as the leukocyte count was only 22.7 × 10E9/L, and no anaemia or thrombocytopenia was found. In the bone marrow, a 65–70% lymphatic infiltrate with slight enhancement in the proportion of prominent, paraimmunoblast-type cells was seen, the overall cellularity being 45%. The peripheral blood cytogenetic analysis revealed 12-trisomy and translocation t(4;12), whereas no signs of p53 deletion in chromosome 17, the ATM gene deletion in 11q, or deletion 13q were found by FISH-analysis.

In 2004, the haemoglobin gradually decreased from a level of above 130 g/L to 110 g/L without increase in lymphocytosis or signs of hemolysis but with mild thrombocytosis. The serum iron content and the transferrin saturation were low (6.5 umol/L and 9%, resp.), and when checked in August, one out of three fecal tests positive for occult blood. Folic acid (214 nmol/L) and B12-vitamin (380 pmol/l) were within normal range. Serum albumin (40.0 g/L) and ionized calcium (1.18 mmol/L/pH7.4) did not suggest that the patient would have had malabsorption. Some fluctuation in INR value was found in 2004, but the value was most of the time within the therapeutic range. The transaminases and plasma creatinine were normal. Because the haemoglobin remained abnormal (114–117 g/L) and also microcytosis developed, endoscopies were arranged to assess the possibility of gastrointestinal bleeding. No symptoms suggesting a gastrointestinal cause for the anaemia existed.

In the gastroscopy, a sliding hernia but no signs of any sources for upper gastrointestinal bleeding were found. The gastroscopy biopsies showed chronic gastritis in the antrum and in the corpus. No active gastritis, atrophic gastritis, *H. pylori, or duodenal villous atrofy* were found. Also, ileocolonoscopy was performed with colonic diverticulosis as the diagnosis. Otherwise, the macroscopic view appeared normal both in the colon and in the ileum.

**Figure 1:**
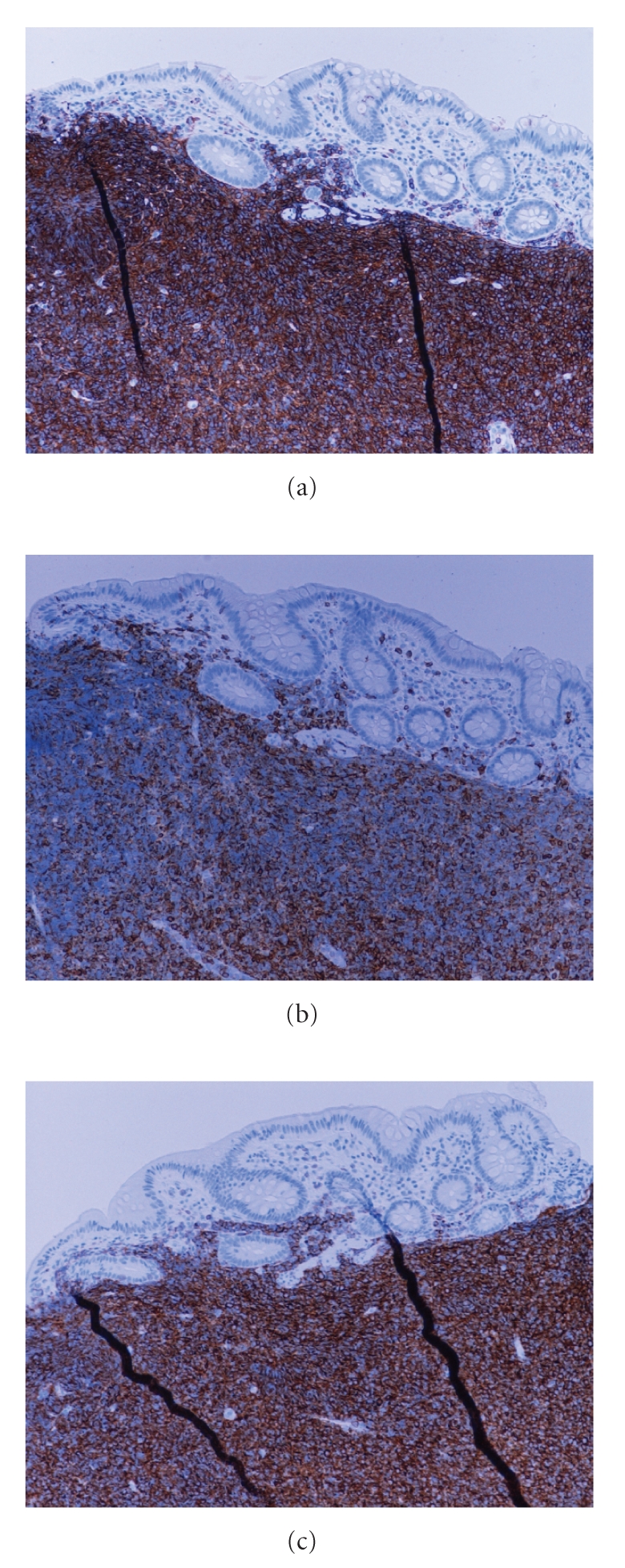
Biopsy specimens revealed multiple diffuse CD20 positive (a) B-cell infiltrates involving mucosa and submucosa of colon without formation lymphoepithelial lesions. B-lymphocytes expressed also CD5 with low intensity (b) and CD23 (c), characteristic of small lymphocytic lymphoma (CLL).

Microscopically dense mucosal and submucosal lymphocytic infiltrates were detected in ileum as well as 4/7 biopsies of colon (Figures 1(a), 1(b), and 1(c)), with predominance of small CD20 positive lymphocytes. No lymphoepithelial lesions were observed. The cells stained positively also for CLL-associated antigens CD5 (low intensity), CD23, and CD43, but were negative for mantle cell-associated antigen cyclin D1. Consecutively, ZAP-70 was positive, and no immunohistochemical staining for CD38 or p53 was detected.

## 3. PATIENT'S OUTCOME, CURRENT TREATMENT

After the endoscopies, the patient was put on peroral iron substitution. After four months, the haemoglobin level had increased to 137 g/L, and the patient experienced an improvement in his general condition. Haemoglobin has thereafter remained within normal range until summer 2006. The most likely reason for the anaemia in 2004 was the blood loss from the GI track and the histologically confirmed CLL GI-manifestation was the underlying reason. Patient has had no GI symptoms like melena, bloody stools, or stomach pain and because the anaemia disappeared with the iron substitution, no other endoscopic procedures like capsule endoscopy or radiological imaging of the small bowel have been assessed. During last two years, haemoglobin has slowly decreased (144–127) and leukocyte level has increased due to the advance of CLL. Despite adverse trisomy 12 karyotypic and ZAP-70+ immunophenotypic findings, the clinical condition and the blood lymphocytosis have continuously remained quite stable, and no active treatment for CLL has been initiated.

## 4. DISCUSSION

Gastrointestinal (GI) CLL involvement is uncommon, and the rare GI complications generally occur only after transformation of CLL to diffuse large B-cell lymphoma (Richter syndrome). Our patient case shows that intestinal manifestations can appear even without any symptoms and without macroscopic signs of CLL in the GI tract. Endoscopies including biopsies are necessary in excluding possible gastrointestinal CLL manifestations. They should be performed especially for patients having GI symptoms or anaemia.

Recent findings suggest that mantle cell lymphoma has a much higher incidence of colonic presentation than previously reported [2]. It seems to appear mostly in asymptomatic patients and is detected microscopically in 50% of patients in a biopsy of a visually benign mucosa. Also, patients with marginal zone B-cell lymphomas have a higher incidence of colonic involvement than previously described. Both of these lymphomas express the mucosal homing receptor *α*4*β*7 especially when situated in the intestine [2–5]. Also, primary follicular lymphoma of intestine is positive for *α*4*β*7 in contrast to nodal follicular lymphoma [6]. Much less is known about the incidence of colonic manifestations of CLL/small lymphocytic lymphoma.

CLL may cause upper GI haemorrhage by directly infiltrating the gastroesophageal junction or through bleeding from oesophageal varices caused by CLL-associated splenomegaly and portal hypertension [7, 8]. According to one case report, protein-losing enteropathy may be found in CLL patients [9]. Reports also mention gastrointestinal CLL manifestations such as infiltration of the intestinal mucosa in the small bowel as well as CLL presenting as colitis [10–12]. CLL, especially after Richter transformation, can cause signs and symptoms suggestive of chronic inflammatory bowel disease [12].

It has been reported that CLL sometimes occurs concomitantly with other malignant neoplasms including melanoma, basal cell carcinoma, laryngeal carcinoma, and colon carcinoma [13–15]. Both cellular and humoral immune responses are often impaired in CLL patients, and the defective immunity in these patients may have had an etiological role in the reported development and rapid progression of their cancers. In the follow-up of CLL patients, we must, therefore, be aware of the possible existence of a second malignant disease [15, 16].

Gastrointestinal CLL manifestations can also form a route for infectious complications. Sadullah et al. have reported a case, where life-threatening gram-negative sepsis developed in a patient with CLL in association with postchemotherapy neutropenia [17]. In colonoscopy, they found a bacterial typhilitis or neutropenic enterocolitis, which is a well described entity of bowel necrosis seen in immunosuppressed or neutropenic patients. It has also been suggested that the small intestinal bacterial overgrowth can possibly contribute to the lymphoid infiltration of the gastrointestinal mucosa in CLL patients [18]. Other rare described CLL manifestations include intussusception and even perforation of the colon [19, 20].

The histopathologic differential diagnosis of common benign lymphatic hyperplasias and various malignant lymphoid disorders of intestine may be challenging, and biopsy specimens should be subjected to rigorous analysis including immunophenotypic and possibly genotypic studies. Abundant biopsy specimens are required to avoid sampling error and to provide for adequate diagnostic tissue material, which should be sent fresh without fixative to haematopathology laboratory. Fresh biopsy specimens can usually be successfully subjected to flowcytometric analysis of immunophenotype and specifically immunoglobulin light chain restriction. Additionally, B-cell and T-cell receptor gene rearrangement studies may be helpful in detecting clonal lymphocyte populations. Immunohistochemical staining of tissue sections enable correlation of the immunophenotype to morphologic features. The diagnostic range for CLL should include CD20+/CD5+ coexpression with CD23+ phenotype, and negative staining pattern for Cyclin D1 to exclude mantle cell lymphoma (lymphomatous polyposis). Differential diagnosis of indolent CD5 negative B-cell lymphomas include follicular lymphoma, which usually has CD10+ phenotype, whereas mucosa-associated marginal zone-lymphoma (MALT-lymphoma) lacks specific phenotypic markers and its immunophenotypic diagnosis is mainly based on exclusion. Lymphoepithelial lesions and plasmacytic differentiation are suggestive of MALT-lymphoma.

In conclusion, also gastrointestinal evaluation should perhaps be part of a complete assessment of the treatment response and remission status in CLL patients in whom the colon was originally involved. If a CLL patient has any symptoms suggesting a possible GI manifestation of the haematologic disease or anaemia not explained by bone marrow infiltration, or hemolysis, the diagnostic evaluation should include endoscopies with adequate biopsies.
